# Periapical Cemento-Osseous Dysplasia in a Medically Compromised Patient: A Case Report

**DOI:** 10.7759/cureus.39623

**Published:** 2023-05-29

**Authors:** Bader Fatani, Abdulaziz G Alotaibi, Yazeed Alzahrani, Mohammed I Almahmoud

**Affiliations:** 1 Dentistry, College of Dentistry, King Saud University, Riyadh, SAU

**Keywords:** periapical cemento-osseous dysplasia, systemic disease, treatment, osseous dysplasia, medically compromised

## Abstract

A fibro-osseous lesion is a condition where the regular bone is changed with a fibrous connective tissue matrix that includes an abnormal bone or cementum. These lesions are divided into three groups: ossifying fibroma, cemento-osseous dysplasia (COD), and fibrous dysplasia. COD is the most recurring benign fibro-osseous lesion. These lesions are usually not detected unless infected and are commonly noted accidentally on an X-ray. In this report, we demonstrate a case of periapical cemento-osseous dysplasia in a medically compromised patient with multiple systemic diseases.

## Introduction

A "benign fibro-osseous lesion" describes a non-cancerous disorder in which regular bone is exchanged by a fibrous connective tissue matrix including cementum or aberrant bone [1]. Based on the pathological, radiological, and clinical characteristics, benign fibro-osseous lesions are divided into three categories: cemento-osseous dysplasia (COD), ossifying fibroma, and fibrous dysplasia. COD is the most frequent benign fibro-osseous lesion, and the mandible is the primary location of this lesion [1]. According to the site of the lesion in the jaw, COD can be categorized into three types: florid, focal, and periapical. Periapical COD arises in the anterior area of the mandible. Focal COD defines lesions that appear in one quadrant of the mandible. Florid COD is an extensive lesion that occurs in more than one quadrant [1]. CODs commonly affect women in their 40 and 50 years [2]. Most of these lesions are asymptomatic unless infected and are frequently seen incidentally on a radiograph [3]. In this report, we demonstrate a case of periapical cemento-osseous dysplasia in a medically compromised patient with multiple systemic diseases.

## Case presentation

**Figure 1:**
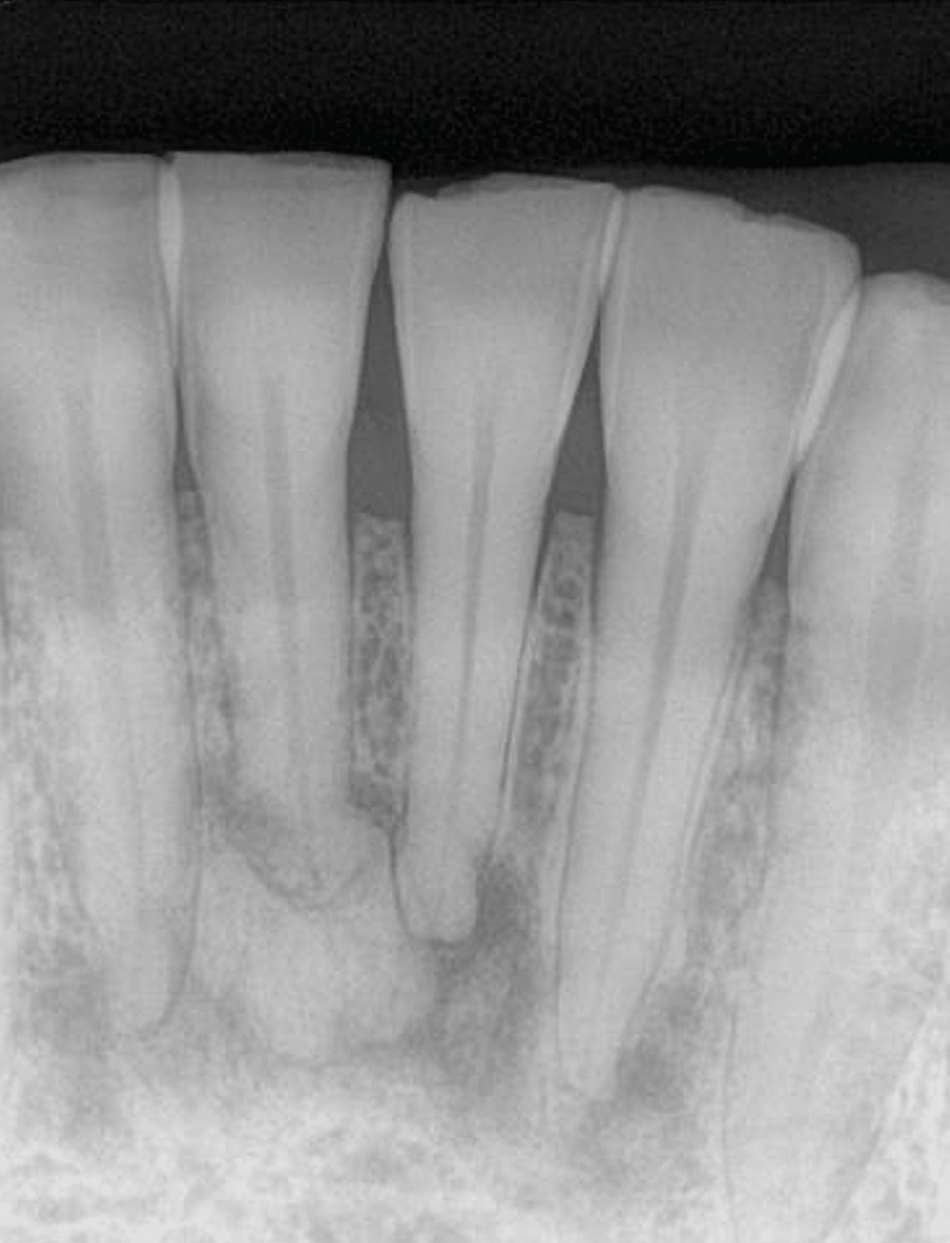
Mixed radiolucency and radiopacity periapical lesion.

A 51-year-old Middle Eastern female patient with controlled hypertension, diabetes type II, hyperlipidemia, and hypothyroidism came to the clinic complaining of delayed wound healing. The onset of these systemic diseases was reported to be more than five years ago. The patient reported using nebivolol (5 mg), gliclazide (60 mg), levothyroxine sodium (50 μg), atorvastatin (20 mg), sitagliptin phosphate (50 mg), and metformin hydrochloride (HCl) (1,000 mg). Extra-oral examination revealed no abnormal symptoms. The patient exhibited a severe dry mouth with a lichenoid plaque on the dorsal of the tongue. Further examination revealed lichen planus and Sjögren's syndrome. Radiographic examination revealed mixed irregular radiolucency and radiopacity apical to the lower anterior teeth. Figure 1 demonstrates the periapical lesion associated with lower incisors.

No tenderness, pain, or swelling was associated with the lesion. The patient had no history of trauma to the mandible. Intra-oral examination showed no expansion in the soft tissue, normal oral mucosa, normal periodontal tissues, and regular teeth color. The lower incisors were asymptomatic, and there was no tenderness or pain on palpation or percussion. Moreover, the lower incisors were vital in an electric pulp test. Multiple differential diagnoses were included, such as cementoblastoma, periapical cyst, and periapical cemento-osseous dysplasia. After a six-month follow-up with the patient, the periapical lesion showed no signs of an inflammatory process or cystic formation. No biopsy was needed due to no inflammatory changes. The patient is scheduled for further periodic observation with periapical and panoramic follow-up to evaluate any possible changes. Oral hygiene instructions were given to the patient to prevent any further infection.

## Discussion

**Figure 2:**
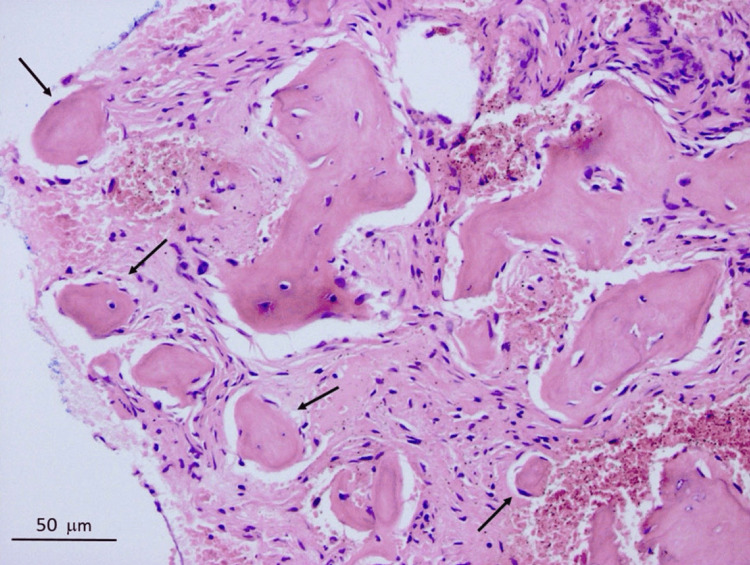
Histological image of cemento-osseous dysplasia. The arrows illustrate acellular segments of cementum-like substances in the loose fibro-collagenous stroma. No osteoclastic activity is shown in the unmarked lamellar bony formations (H&E: ×400). © The Author(s). 2019 Open Access. This article is distributed under the terms of the Creative Commons Attribution 4.0 International License (http://creativecommons.org/licenses/by/4.0/), which permits unrestricted use, distribution, and reproduction in any medium, provided you give appropriate credit to the original author(s) and the source, provide a link to the Creative Commons license, and indicate if changes were made. The Creative Commons Public Domain Dedication waiver (http://creativecommons.org/publicdomain/zero/1.0/) applies to the data made available in this article unless otherwise stated [7]. H&E: hematoxylin and eosin

The ideal approach to diagnosing COD is based on clinical and radiological findings [1,3-6]. The majority of these lesions are asymptomatic, frequently found by accident on a radiograph, and do not require any treatment [3-5,7]. Biopsies should only be conducted in limited cases [1]. Figure 2 demonstrates a histological image of cemento-osseous dysplasia as reported in the literature.

Studies have shown that COD primarily affects women aged 40-50 years, with the highest rate at age 40-69 years, especially African-Americans followed by Asian women [1,3-7]. To this day, there is no known etiology for COD [1]. Trauma caused by occlusion, caries, periodontal disease, infectious diseases, hormonal imbalance, and systemic diseases is thought to be causative factors [5]. The type of COD can be classified clinically into three categories based on location: posterior (focal), anterior (periapical), and more than one quadrant (florid) [1,5,8]. The most prevalent type of the three types is not clear [1]. Based on developmental origin, it can be classified into reactive or neoplastic origins [6]. A biopsy is the only conclusive way to distinguish COD from other bone lesions [5]. To prevent lesion infection, patients with COD should undergo periodic observations and receive routine panoramic follow-ups to assess any further changes [1,4,5]. Oral hygiene should also be maintained even if they are asymptomatic. Generally, treatment is unnecessary for the nature of this lesion [4,6,7]. However, if symptoms were observed, then surgery is usually required [1,7]. Although there is uncertainty over the best course of treatment for COD lesions, curettage/surgical removal, either with or without antibiotics or analgesics, was reported to be the most appropriate treatment [1].

The most common COD subtypes associated with infection were florid COD (FCOD) (62.1%), focal COD (27.3%), and periapical COD (10.6%) [2]. A study of 66 cases of infected COD showed that females were more likely than males to have an infection within a COD lesion. There were 21:1 more women than men. The mean age of the affected persons was 57.4 years (± 10.1) at the time of diagnosis (range: 40-83 years) [2]. Due to reduced vascularization, increased bone hardness, and acellularity, infections that develop within the COD are more likely to cause necrosis. Chronic inflammatory periodontal disease, tooth decay that can result in pulp necrosis, tooth extraction, and minor irritation from dentures are some potential sources of infection [1]. Focal COD lesions need to be identified separately on conventional X-rays from other hardened lesions that may look similar. Paget's disease causes a fluffy appearance involving several bones such as the jaw, skull, and pelvis and leads to high alkaline phosphatase levels. On the other hand, focal COD is focused above the inferior alveolar canal in the jaw and does not raise serum alkaline phosphatase levels above the normal range [9]. A previous study by Bastos et al. [10] explained some of the clinicopathological features of florid cemento-osseous dysplasia-related osteonecrosis. The authors showed that FCOD-related osteonecrosis is more commonly found in female adults, particularly in the mandibular area, and that histopathological features such as osteomyelitis, bone resorption, and bacterial colonization are more frequently observed in these cases [10]. As explained by Urs et al. [11], the features seen under a microscope in COD can resemble those of other fibro-osseous lesions. Therefore, it is crucial to examine and compare clinical, radiographic, and histopathological aspects to achieve an accurate diagnosis of COD. Moreover, careful pulp testing and familiarity with the common locations, radiographic presentations, and non-threatening behavior of periapical COD can help prevent misdiagnosis and avoid unnecessary treatment [12].

## Conclusions

Systemic diseases are one of the causative factors of periapical cemento-osseous dysplasia. In this paper, we report a case of periapical cemento-osseous dysplasia in the anterior mandible associated with multiple chronic systemic diseases, including controlled hypertension, diabetes type II, hyperlipidemia, and hypothyroidism. Periapical cemento-osseous dysplasia is usually asymptomatic and benign in origin. However, careful intra-oral examination and evaluation of past medical history are essential to detect COD and prevent further infection.
